# Sense of Coherence in Healthcare Workers During the COVID-19 Pandemic in Ecuador: Association With Work Engagement, Work Environment and Psychological Distress Factors

**DOI:** 10.3389/ijph.2022.1605428

**Published:** 2022-12-05

**Authors:** Juan Gómez-Salgado, Cristian Arturo Arias-Ulloa, Mónica Ortega-Moreno, Juan Jesús García-Iglesias, Kenny Escobar-Segovia, Carlos Ruiz-Frutos

**Affiliations:** ^1^ Department of Sociology Social Work and Public Health, Faculty of Labour Sciences, University of Huelva, Huelva, Spain; ^2^ Safety and Health Postgraduate Programme, Universidad Espíritu Santo, Guayaquil, Ecuador; ^3^ Faculty of Engineering in Mechanics and Production Sciences, Escuela Superior Politécnica del Litoral, Guayaquil, Ecuador; ^4^ Department of Economy, University of Huelva, Huelva, Spain; ^5^ Faculty of Engineering in Earth Sciences, Escuela Superior Politécnica del Litoral, Guayaquil, Ecuador

**Keywords:** COVID-19, health personnel, psychological distress, sense of coherence, work engagement

## Abstract

**Objectives:** The aim of this study was to test the association between the sense of coherence, work engagement, and psychological distress in healthcare workers in Ecuador during the first phase of the COVID-19 pandemic.

**Methods:** A cross-sectional observational study in a sample of 803 healthcare professionals from all regions of Ecuador between 2 April and 17 May 2020. A self-administered questionnaire was used, which contained sociodemographic and work environment variables, the Utrecht Work Engagement Scale (UWES-9), the General Health Questionnaire (GHQ-12), and Sense of Coherence Scale (SOC-13).

**Results:** The mean value of sense of coherence was M = 65.04; SD = 12.74; for work engagement, it was M = 39.36; SD = 10.53; and for psychological distress, M = 4.58; SD = 3.44. There is a positive correlation (*p* < 0.01) between the sense of coherence and work engagement, and a negative correlation with psychological distress.

**Conclusion:** During the pandemic in Ecuador, healthcare professionals have suffered a major deterioration of their mental health. Sense of coherence has been associated with work engagement and psychological distress. They have perceived a worsening of the quality of care and working conditions compared to those existing before COVID-19**.**

## Introduction

COVID-19, which originated in Wuhan, China as an atypical pneumonia [1], brought about an overload in health systems in all countries, especially in Intensive Care Units in Europe [2] and in less economically developed countries, such as Ecuador, with a lack of supplies, personnel, and installed capacity [3]. This situation did not only lead to biological pathologies, but also the psychological distress (PD) of healthcare professionals increased [4].

A multicentre study found that the COVID-19 pandemic was associated with a higher incidence of mental health symptoms than that identified in previous stressful situations such as post-traumatic stress disorder, anxiety, depression, insomnia, and dissociation, being higher in Latin America and lower in North America [5].

Healthcare workers are being the most studied group internationally in relation to the COVID-19 pandemic, although there is still a gap in the literature regarding organisational support aimed at the mental health of healthcare workers [6], especially concerning professionals in charge of treating infected patients [7]. PD has been proven to be linked with patient safety and care, family and work environment, media and public perceptions, and government response to the pandemic, with exacerbations of uncertainty, hypervigilance, and moral distress found to increase the level of PD [8].

Ecuador is a country of about 17 million people with an uneven distribution of the health system to deal with severe cases of the disease, especially in the coastal areas, where COVID-19 cases have overlapped with a high number of dengue cases. In addition, it is considered that there is no universal health coverage because of transport difficulties and geographical issues. Therefore, some groups of indigenous population or refugees found it more difficult to receive appropriate health attention [ [9]]. This situation is similar to that of many other Latin American countries and differs from the lower number of difficulties faced by European countries and the USA in diagnosing and controlling the pandemic [10]. Ecuador is a country where one of the largest COVID-19 pandemic health scares in Latin America occurred, especially in more populated and industrialised areas such as Guayaquil [11], which accounted at certain times for 70% of all reported cases in the country [12]. This revealed that the response of the Ecuadorian health system in the early stages of the pandemic was not as rapid and effective as might have been expected [13].

Compared to studies in Asia, the USA, or Europe, there are few articles assessing the PD of healthcare workers in Latin American countries [14–16]. In these cases, stress, anxiety, depression, and post-traumatic stress reached significantly high levels of incidence [17]. In particular, distress has been described as especially high in front-line healthcare professionals who worked with COVID-19 patients [18].

Work engagement (WE), measured through the Utrecht Work Engagement Scale (UWES) is a positive and satisfying work-related attitude defined by the vigour, dedication, and absorption dimensions [19]. It is a multi-axial concept that brings together multiple factors that influence WE, including organisational climate; work, professional, and personal resources; job demands; and demographic variables [20]. Sense of coherence (SOC) is described as an ability to understand a situation, perceive it as manageable, and mobilise resources to develop an effective response, and is composed of the comprehensibility, manageability, and meaningfulness dimensions [21].

It is known from previous studies that sense of coherence and work engagement are key influencing factors for healthcare workers [22] and that lower SOC may be a protective factor in later stages of the pandemic [2]. Work engagement and sense of coherence positively correlated with each other and both negatively with PD. Thus, healthcare professionals, though experiencing PD, perceive their work positively and satisfactorily despite the severity of the situation and the harsh working conditions [23]. Looking specifically at women, both healthcare and non-healthcare workers, the presence of work overload and concerns about their health status or economic situation were observed, and these variables were predictors of stress among these workers in the second wave of the pandemic [24].

The importance of maintaining an optimal work environment has been shown not only to increase workers’ motivation, satisfaction, or performance [25], but also to reduce the negative effects of the pandemic on workers’ mental health [26]. It was found that, among nurses, the increased workload of the pandemic was positively associated with work engagement [27].

Sense of coherence, according to the salutogenic model [28, 29], is known to be an important predictor and modulator of mental health and psychopathological symptoms during the pandemic, and these changes are sustained over a long period of time [30]. Prior to the COVID-19 pandemic, it had been proven that a high SOC in nurses was associated with better health and work engagement [31]. In non-healthcare workers who performed essential activities during the confinement period of the first phase of the pandemic, it was observed that low levels of WE and SOC were associated with higher levels of PD [32].

The purpose of this research was to test the association between the sense of coherence, work engagement, work environment, and psychological distress in healthcare workers in Ecuador during confinement in the first phase of the COVID-19 pandemic.

## Methods

### Study Design

The study design was descriptive cross-sectional.

### Participants

According to data from 2019, the global number of healthcare professionals in Ecuador was about 90,000, with 39,593 physicians, 25,483 nurses, 17,221 nursing assistants, 5,508 dentists, 1,615 clinical psychologists, and 2,278 midwives [33]. A total of 1,235 healthcare professionals from all provinces (regions) of Ecuador participated, yet with higher percentages from the province (region) of Pichincha (31.2%) and Guayas (24.5%). After eliminating questionnaires that were not 99% completed, 803 (65%) were finally incorporated. The criteria for inclusion in the research were: 1) being an active healthcare professional; 2) over 18 years of age; and 3) living in Ecuador during the COVID-19 pandemic.

### Measuring Instruments

A self-elaborated questionnaire based on similar studies of other pandemics was applied for data collection [34] (Supplementary Material). Different sources were used: socio-demographic data (sex, age, marital status, educational level, children, pet, type of work) and work environment (Table 1). The categorisation of those variables was related to the work environment, with scores between 1 and 10 [14], sense of coherence (SOC) [29], Utrecht Work Engagement Scale (UWES) [35], and Goldberg’s General Health Questionnaire [36]. They were asked to assess the changes in the quality of care, working conditions, occupational health, and patient safety pre and post-pandemic. Scoring of the variables was established out of 10.

**TABLE 1:** Pandemic-related work environment questions (Ecuador, 2020).

Variable	Question
Effectiveness	Do you think your department, service, unit or company has provided you with the necessary means and material to EFFECTIVELY carry out your job?
Safety	Do you think your department, service, unit or company has provided you with the necessary means and material to SAFELY carry out your job?
Distance	Do you consider appropriate the distance maintained with your work mates?
Contact	Are you in contact with clients/users/patients that could be a source of risk?
Conflict	Have you observed any increase in labour conflict in your job?
Risk	Do you think your profession or workplace put you at risk of getting infected?
Acceptance	Do you accept the risk of getting infected as part of your job?
Psycho1	Do you believe it would be important to offer psychological support to professionals and volunteers who are actively taking part in the COVID-19 health crisis?
Psycho2	Do you believe it would be important to offer psychological support to persons and their families who are directly affected by COVID-19 to deal with the difficulties arisen from the health crisis?
Psycho3	Do you believe it would be important to offer psychological support to the general population to deal with the difficulties arisen from the COVID-19 health crisis?
Workload	Do you consider there has been an increase in the workload after the onset of the health crisis?
Stress	Do you feel more stressed at work?
Satisfaction	How would you score your job satisfaction during the present COVID-19 situation?
Appreciation	As a healthcare professional, do you feel appreciated by society?

Scoring of the variables out of 10, being 1 the least favourable and 10 the most favourable.

SOC was measured with the SOC-13 scale, a 13-item questionnaire with a Likert-type response range from 1 to 7, where 1 is least frequent and 7 is most frequent. The score range of the scale can vary from 13 to 91, with a lower score indicating a low level of SOC, and it has 3 dimensions: meaningfulness, comprehensibility, and manageability. A Cronbach’s alpha index of 0.808 was calculated, considering the whole instrument. The internal consistency indices presented by the different dimensions were α = 0.652 for comprehensibility, α = 575 for manageability, and α = 0.570 for meaningfulness [28, 29, 37–39].

To assess the WE, the UWES-9 was used. This questionnaire consists of 9 questions, with the highest score being 54 and indicative of high WE. It has a Likert-type response range from 0 (never) to 6 (always), distributed in 3 dimensions: Vigour, Dedication, and Absorption. The internal consistency for the complete questionnaire was α = 0.928, being α = 0.855 for Vigour, α = 0.852 for Dedication, and α = 0.757 for Absorption [35].

PD was measured with the Goldberg’s General Health Questionnaire (GHQ-12). This tool is designed to assess mental health through 12 questions or items, using a Likert-type response range from 1 to 4, taking as 0 the answers scored as 1 or 2, and as 1 the answers scored as 3 or 4, and assuming an overall score from 0 to 12 points. The total score was calculated by adding the scores obtained in all items of the dichotomous scale and 3 was considered a breakpoint for this one-dimensional screening instrument (Cronbach’s α = 0.874) [36].

The entire questionnaire was validated in Spain by a group of experts [14] and then culturally adapted to the population of Ecuador to ensure good understanding of the items and to include country-specific data.

### Procedure

Non-probability snowball sampling was used, sending the questionnaire through social networks and through the channels of various public institutions and universities. The questionnaire was distributed through the Qualtrics^®^ online platform to staff of health institutions and scientific associations. An invitation to participate was sent by e-mail, including a link to access the questionnaire. Participants were invited to share the questionnaire with their colleagues, following a snowball sampling effect. The information was filled in through different electronic media with internet access. Data collection took place between 2 April and 17 May 2020.

### Data Analysis

Absolute frequencies and percentages were presented for the different categories of socio-demographic variables, and mean values and standard deviations were collected for the SOC variable in each of them. The t-student test for independent samples allowed to contrast the existence or absence of differences in SOC between categories; in order to establish the difference in perception of the pre and post-pandemic situation, t-student test was used for the related samples; and, finally, the effect size was assessed with Cohen’s d. For the quantitative variables of interest in the study, descriptive measures were provided (mean, standard deviation, skewness, kurtosis, minimum, and maximum) and correlations were studied with Spearman’s Rho coefficient.

A multiple linear regression model for SOC was presented for those variables that were significantly correlated. The model was validated through the ANOVA test; the normality of the standardised residuals was studied with the Kolmogorov-Smirnov test; multicollinearity was assessed based on the tolerance and the variance inflation factor (VIF), selecting a model with a maximum condition index of less than 20, i.e. the limit established by Belsley. The hypotheses of linearity of the independent variables and homoscedasticity of the residuals were tested graphically, and the independence of the residuals was tested with the Durbin-Watson statistic.

Finally, a regression tree (CART) was built for the SOC with cases from the sample to detect relationships of interest. Optimal cut-off points were selected for improvement so that cases in each part were similar to each other and different from cases in any other part. The nodes showed the mean values of the group and the percentage of cases in the node over the total sample. This method allowed to predict the percentage of those suffering from PD in new cases. The tree was validated by sample splitting. Analyses were carried out using SPSS 26.0 and R statistical software, version 4.0.0.

### Ethical Considerations

The study was authorised in Ecuador by the Research Ethics Committee of the Universidad San Gregorio de Portoviejo (USGP-DI-049-2021) and in Spain by the Research Ethics Committee of the Health System in Huelva, belonging to the Regional Ministry of Health of Andalusia, Spain (PI 036/20).

## Results

### Socio-Demographic Variables in Relation to the Sense of Coherence

The sample had a mean age of 33.8 years, with a standard deviation of 8.13 years, within an age range of 18–70 years. The majority were women (65.3%), not living with a partner (56.9%), without children (52.2%), with pets (57.2%), and mostly with university education (95.6%) and working outside from home during the pandemic (76.6%) (Table 2). Table 2 shows a statistically significant difference in the SOC, which is higher among those with a partner and those with children, *p* <0.05.

**TABLE 2:** Socio-demographic variables versus Sense of Coherence (Ecuador, 2020).

	N (%)	SOC-13	Independent t-tests (Sig.)	Cohen’s d
		M (SD)		
Sex				
Male	279 (34.7)	65.74 (12.56)	1.130 (0.259)	0.084
Female	524 (65.3)	64.67 (12.82)		
Marital status				
With a partner	346 (43.1)	66.29 (13.29)	2.421 (0.016)	0.173
Without a partner	457 (56.9)	64.10 (12.23)		
Educational level				
Upper secondary school or lower	35 (4.4)	66.89 (14.72)	0.762 (0.451)	0.151
University or higher	768 (95.6)	64.96 (12.64)		
Children				
Yes	384 (47.8)	66.25 (12.73)	2.582 (0.010)	0.182
No	419 (52.2)	63.94 (12.66)		
Pet				
Yes	459 (57.2)	65.21 (13.11)	0.429 (0.668)	0.031
No	344 (42.8)	64.82 (12.23)		
You work				
From home	188 (23.4)	65.78 (12,49)	0.903 (0.367)	0.075
Outside	615 (76.6)	64.82 (12.81)		

N: sample; %: percentage; M: mean; SD: standard deviation.

### Quality of Healthcare, Working Conditions, and Patient Health and Safety Before and After the Pandemic

Table 3 shows the perceived quality of healthcare compared to that existing before the COVID-19 health emergency: M = 5.16 versus M = 6.19. The results obtained for working conditions were M = 5.69 versus previous M = 6.63; perception of occupational health, M = 5.47 versus previous M = 6.61; and patient safety, M = 5.90 versus previous M = 6.86, all differences being statistically significant *p* <0.01.

**TABLE 3:** Perception of pre and post-pandemic variation in quality of care, working conditions, occupational health, and patient safety (Ecuador, 2020).

	Quality of care		Working conditions		Occupational health		Patient safety	
	Before the health emergency	Currently/During the health emergency	Before the health emergency	Currently/During the health emergency	Before the health emergency	Currently/During the health emergency	Before the health emergency	Currently/During the health emergency
N	803	803	803	803	797	803	786	803
Mean	6.19	5.61	6.63	5.69	6.61	5.47	6.86	5.90
SD	2.11	2.48	2.10	2.45	2.26	2.57	2.05	2.61
Skewness	−0.555	−0.182	−0.668	−0.241	−0.517	−0.102	−0.575	−0.217
Kurtosis	−0.035	−0.823	0.139	−0.777	−0.327	−0.891	−0.107	−0.935
Paired t-tests (Sig.)	8.032 (<0.001)		11.783 (<0.001)		13.583 (<0.001)		10.389 (<0.001)	
Cohen’s d	0.283		0.416		0.479		0.371	

Scoring of the variables out of 10.

### Sense of Coherence, Work Engagement, Psychological Distress, and Correlations Between Variables

Table 4 shows the mean value of sense of coherence is M = 65.04, where the dimension comprehensibility is M = 23.90 and manageability M = 18.90. The UWES mean is M = 39.36, with dimension dedication M = 13.77 and absorption dimension, M = 13.55. The mean value of PD was M = 4.58.

**TABLE 4:** Results of the Sense of Coherence Scale, Work Engagement Scale, and General Health Questionnaire, and their respective dimensions or variables, during the COVID-19 pandemic, and correlations between variables (Ecuador, 2020).

	N	Mean	SD	Skewness	Kurtosis	Minimum	Maximum	*Spearman*’*s Rho*													
								SOC_13	Age	UWES	GHQ_12	Effectiveness	Safety	Distance	CONFLICT	PSYCHO1	PSYCHO2	PSYCHO3	Stress	Satisfaction	Appreciation
SOC_13	803	65.04	12.74	−0.26	−0.32	19	91	—													
AGE	797	33.8	8.13	1.76	3.51	18	70	0.171 (<0.001)	—												
UWES	803	39.36	10.53	−0.74	−0.05	2	54	0.439 (<0.001)	0.089 (0.012)	—											
GHQ_12	803	4.58	3.44	0.47	−0.81	0	12	−0.483 (<0.001)	−0.013 (0.715)	−0.422 (<0.001)	—										
Effectiveness	803	6.41	2.90	−0.50	−0.89	1	10	0.202 (<0.001)	−0.042 (0.240)	0.266 (<0.001)	−0.235 (<0.001)	—									
Safety	803	6.42	2.94	−0.48	−0.95	1	10	0.192 (<0.001)	−0.048 (0.177)	0.255 (<0.001)	−0.223 (<0.001)	0.923 (<0.001)	—								
Distance	615	6.73	2.67	−0.59	−0.60	1	10	0.169 (<0.001)	0.071 (0.080)	0.217 (<0.001)	−0.116 (0.004)	0.416 (<0.001)	0.425 (<0.001)	—							
CONFLICT	803	6.21	3.01	−0.42	−1.07	1	10	−0.186 (<0.001)	0.023 (0.512)	−0.148 (<0.001)	0.229 (<0.001)	−0.114 (0.001)	−0.093 (0.008)	−0.031 (0.440)	—						
PSYCHO1	803	9.25	1.66	−2.62	6.89	1	10	0.106 (0.003)	0.053 (0.133)	0.029 (0.417)	0.076 (0.031)	0.011 (0.745)	0.023 (0.515)	0.055 (0.172)	0.095 (0.007)	—					
PSYCHO2	803	9.42	1.38	−2.89	8.84	1	10	0.115 (<0.001)	0.046 (0.196)	0.062 (0.080)	0.032 (0.365)	0.028 (0.422)	0.030 (0.399)	0.079 (0.049)	0.069 (0.050)	0.744 (<0.001)	—				
PSYCHO3	803	9.19	1.52	−2.11	4.20	1	10	0.124 (<0.001)	0.065 (0.069)	0.083 (0.019)	0.045 (0.203)	0.049 (0.164)	0.054 (0.129)	0.082 (0.041)	0.092 (0.009)	0.591 (<0.001)	0.671 (<0.001)	—			
Stress	803	7.91	2.56	−1.23	0.58	1	10	−0.176 (<0.001)	−0.027 (0.443)	−0.265 (<0.001)	0.357 (<0.001)	−0.122 (<0.001)	−0.099 (0.005)	−0.085 (0.035)	0.405 (<0.001)	0.296 (<0.001)	0.241 (<0.001)	0.281 (<0.001)	—		
Satisfaction	803	6.57	2.44	−0.55	−0.36	1	10	0.244 (<0.001)	−0.033 (0.346)	0.371 (<0.001)	−0.263 (<0.001)	0.322 (<0.001)	0.312 (<0.001)	0.212 (<0.001)	−0.070 (0.047)	−0.011 (0.753)	0.021 (0.550)	0.059 (0.092)	−0.091 (0.010)	—	
Appreciation	786	6.63	2.66	−0.56	−0.58	1	10	0.297 (<0.001)	0.071 (0.048)	0.342 (<0.001)	−0.271 (<0.001)	0.281 (<0.001)	0.302 (<0.001)	0.205 (<0.001)	−0.054 (0.130)	0.037 (0.302)	0.076 (0.034)	0.093 (0.009)	−0.102 (0.004)	0.420 (<0.001)	—

N: sample; SD: standard deviation. SOC_13: Sense of Coherence Scale; UWES: Utrecht Work Engagement Scale; GHQ-12: Goldberg’s General Health Questionnaire. Explanation of the variables in Table 1.

The assessment of the measures taken by the companies to protect themselves against the pandemic had a similar rating, with those of effectiveness, safety, and distance maintained by co-workers being rated between 6 and 7. The lowest score was given to the level of conflict in the workplace M = 6.21 (SD = 3.05). The mean risk of infection at work was M = 8.72. The degree of acceptance of being infected at work was M = 6.38, the degree of satisfaction with their work during the pandemic M = 6.57 and the level of perception of being appreciated as a healthcare professional during this situation M = 6.63. On the contrary, the highest scores were given to the importance of offering psychological support to professionals and volunteers who intervene directly in the health crisis M = 9.25, affected people and families M = 9.42, and the general population M = 9.19, these variables being the ones with the greatest skewness and kurtosis. The mean of people who felt stressed at work was M = 7.91, and for those who had experienced an increase in their workload after the onset of the health crisis, it was M = 7.76.

Table 4 also shows that there is a statistically significant (*p* <0.01) positive correlation between the SOC and WE, and a negative correlation with PD. Similarly, there is a positive correlation (*p* <0.01) between the SOC and the effectiveness provided by the company to perform an effective and safe job, that colleagues keep a safe distance, the degree of satisfaction with their work, and the level of perceived appreciation as a professional by society. There is also a positive correlation (*p* <0.01) between the SOC and age, and between the SOC and the psychological support needs of patients, caregivers, and the general population. In contrast, there is a negative correlation (*p* <0.01) between the SOC and the perception of increased conflict in their work, as well as with the perception of stress at work.

### Multiple Linear Regression Model and Classification Tree Obtained in the Multiple Linear Regression Model

To determine the multiple linear regression model explaining the sense of coherence (SOC), variables with a significant correlation with SOC-13, at the 0.01 level, were considered. Relevant data on the independent variables included in the model is shown in Table 5. Among these, PD stands out with an inverse relationship; age, social esteem, and the WE test score were assessed as with less importance and with a direct relationship. Finally, the increase in work conflict shows an inverse relationship. All of them were statistically significant. The presented model is valid, F-Snedecor = 80.085, with sig. <0.001, and explains 34.1% of the variance of the dependent variable (adjusted R^2^ = 0.337). In addition, the normality of the standardised residuals was studied with the Kolmogorov-Smirnov test, whose value was 0.032, with a significance level of 0.053. Values close to tolerance and VIF values were indicators of non-collinearity. Finally, a Durbin-Watson value of 1.941 proved the independence of the residuals.

**TABLE 5:** Multiple linear regression model (Ecuador, 2020).

Model	Standardised coefficients	95.0% confidence interval for B		Collinearity statistics	
	Beta	Lower limit	Upper limit	Tolerance	VIF
Constant		47.068	57.104		
GHQ-12	−0.349	−1.537	−1.052	0.768	1.302
UWES-9	0.235	0.204	0.362	0.765	1.307
Age	0.129	0.115	0.304	0.975	1.026
Appreciation	0.088	0.128	0.717	0.867	1.153
Conflict	−0.073	−0.558	-0.059	0.946	1.057

Dependent variable: SOC-13. SOC: Sense of Coherence Scale; UWES: Utrecht Work Engagement Scale; GHQ-12: Goldberg’s General Health Questionnaire. Appreciation: As a healthcare professional, do you feel appreciated by society? CONFLICT. Have you observed any increase in labour conflict in your job?

Note: The hypotheses of linearity of the independent variables and homoscedasticity of the residuals were tested graphically.

**FIGURE 1:**
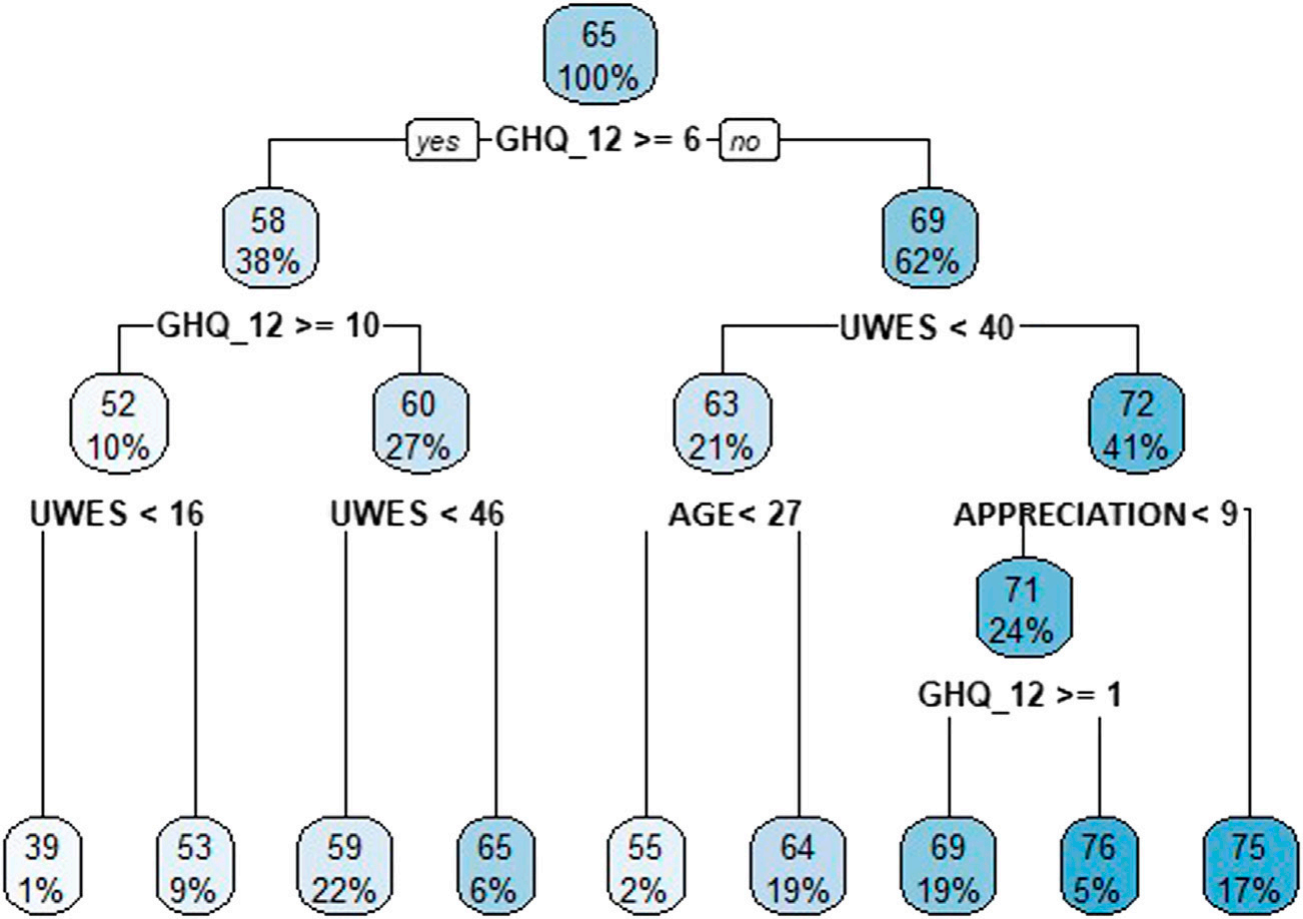
Classification tree based on the variables obtained in the linear regression model (Ecuador, 2020). UWES, Utrecht Work Engagement Scale; GHQ-12, Goldberg’s General Health Questionnaire.

In Figure 1, it is shown how the classification and regression tree for the SOC starts on a root node from which it branches according to PD and WE. For PD values greater than or equal to 9.5, two terminal nodes are distinguished with mean SOC values equal to 38.87, in 1% of the cases with UWES scores below 15.5, and equal to 53.22 points in the other 9%. When the PD score ranges between 5.5 and 9.5, the mean SOC is 58.69 in 22% of the data with UWES score below 45.5 points, and 65.31 points when it is above or equal. For 21% of the sample with a PD below 5.5 and UWES score below 39.5, the mean SOC value is equal to 55.15 in individuals younger than 26.5 years, increasing to 64.44 in individuals older than 26.5 years. Finally, when the PD is lower than 5.5 and the UWES score is greater than or equal to 39.5, the SOC is classified into three terminal nodes; if the level of appreciation by society is lower than 8.5 and the PD is lower than 0.5 points, 5% of the sample is grouped together with a mean sense of coherence of 76.33 points; with higher PD values, 19% of the sample is classified with a mean value of 69.07; and if the appreciation of society reaches values above 8.5, 17% is classified with a mean value of 74.81.

## Discussion

This study has allowed to assess the SOC of healthcare professionals in Ecuador during the first phase of the COVID-19 pandemic, which could have been influenced by the WE, work environment, and PD variables.

The results obtained confirm previous findings in which a high SOC was positively associated with work environment, and both were negatively associated with PD [2, 24]. Work environment is influenced by the level of job satisfaction, where SOC acts as a modulating factor and where both job satisfaction and SOC are better predictors of work environment than resilience [40]. Similarly, the association found between a positive work environment and a high SOC confirms previous studies, which have found that an improvement in the working environment of nurses, with an increase in SOC, leads to improvements in their own health [41].

In line with the present results, it is known from previous studies that SOC and WE are key influencing factors for workers. In this sense, European healthcare workers with higher levels of work-related SOC may have been protected from changes in psychological symptomatology for about 3 months, decreasing this level of protection over the course of the pandemic and leading to the deterioration of mental health [22]. In contrast, healthcare professionals with lower SOC may be protected in later stages of the pandemic [2].

The present study shows that people who have a partner and those who have children have a greater SOC, which confirms previous studies performed in Japanese hospitals where higher levels of PD were found in those living alone [42], thus finding an inverse association between the SOC and PD. On the other hand, this differs when analysing women and younger people [42], or when relating women with the level of studies [31], variables for which no differences have been found regarding the SOC. Having studies on healthcare workers during the COVID-19 pandemic, affecting all workers regardless of their different socio-demographic characteristics [43], should be necessary.

What has not been possible to assess has been whether infected healthcare workers showed differences with non-infected workers, as studies have found differences in their mental health [44]. This has been due to the fact that, although participants were asked about it in the questionnaire, in the early phases of the pandemic diagnostic tests were carried out on a low percentage of professionals, which is consistent with the worsening of occupational health conditions perceived in the present study in that phase of the pandemic and whose effects had been maintained 6 months after the end of confinement [45].

A not so small percentage, 23.4% of the healthcare workers who responded, worked from home. This is the group that in the first phase of the COVID-19 pandemic was involved in administrative activities or did not require contact with the infected, physicians who attended medical or nursing consultations by videoconference or telephone or who managed suspected cases of the disease, among others. No differences were observed between those who teleworked and those who continued working in health centres, probably because there were health risks associated with exposure to the sick and also associated with teleworking and depending on the working conditions or those of their own homes [46–49].

Predictably, healthcare professionals consider that the quality of healthcare, working conditions, and occupational health have worsened significantly during the health crisis, as compared to the previous situation. Previous studies have proposed strategies to enhance positive factors at work that compensate for increased workload and associated stress [50]. Stressors may be associated with the threat of contracting the disease, infecting others, or with work itself, but may also be due to measures taken to limit the transmission of the virus [5]. Socio-economic and political reasons have been offered for why certain countries had a worse response to the pandemic [51], among others the low percentage of healthcare professionals who had been vaccinated in some countries and which may have been influencing their behaviour within the population as a whole [52]. In Ecuador, the public health system’s response seemed insufficient specially regarding human resources and equipment, which increased the exposure to risk and fear of contagion of relatives [53].

The results found in the present study, in which healthcare workers have perceived a clear worsening of their working conditions during the pandemic, are consistent with what has already been published in Ecuador [11, 12] and explainable by the slow response of the Ecuadorian health system [13]. Being able to maintain and not worsen the working conditions in health centres during a pandemic will depend, to a large extent, on the strength of the public health system to take on this unforeseen increase in workload, especially in Primary Health Care [54]. However, as detailed in previous studies, public employees showed higher PD compared to employees of private or independent companies in this country [55]. The need to redefine the public mental health system after the COVID-19 pandemic in developed countries has been raised [56, 57], but this is more difficult for developing countries, such as most Latin American ones.

It should be noted that the data were collected in the first phase of the COVID-19 pandemic, where the peak incidence occurred. This can be compared to the study conducted in four countries (Argentina, Chile, Colombia, and Ecuador) where, as expected, the worst mental health outcomes for healthcare workers were found during the peaks of the highest incidence of cases, in those working in Intensive Care Units, and those who were infected or had doubts about being infected [15].

The COVID-19 pandemic has highlighted the role of public health and the importance of acting in all areas of life: family, community, education, leisure, and work; this would be facilitated by incorporating the “occupational health perspective” into the public health system [58].

Among the limitations of the study, it is worth mentioning that most of the analysed population had university level of studies, as it was focused on medical and nursing staff. Thus, the results are less applicable to less qualified healthcare workers, who should also be studied. Another limitation is not having analysed the results in terms of the job function or whether the participant was working for a public or private institution. Another limitation could have been the need to have devices with internet access, but the group studied did not encounter any problem in this regard, and the questionnaire could be answered from any device: computer, tablet, or mobile phone. Regarding the statistical analysis, the non-probabilistic snowball sampling design, disseminated through institutions and social networks, was chosen to achieve a rapid response in the first phase of the pandemic, being this the method that has subsequently been used in the European [59]. Furthermore, it should also be noted that the internal consistency of the SOC scale in its published version adapted to Spanish obtained low values. On the other hand, the validation of the SOC-13 published in Spanish does not provide values for the minimum clinically important differences (MCID) either [60]. This issue could affect the assessment of the sense of coherence in the present study. Therefore, future studies should update the psychometric properties of the scale in its Spanish adaptation.

The greatest limitation of this research is related to the work requirements of health personnel during the first phase of the pandemic, when the information was collected, conditioning the number of responses but, at the same time, giving greater value to the people who responded. Some variables have not been considered in this study, but it would be necessary to include them in future research, such as the specific area of work, work experience and work status.

In conclusion, during the first phase of the pandemic, the variables that determine the level of sense of coherence among healthcare workers in Ecuador are PD, WE, work environment, age, feeling appreciated by society as a healthcare professional, and having observed an increase in labour conflict in their work during the pandemic.

The healthcare workers assessed felt that the quality of care, working conditions, occupational health, and patient safety had significantly worsened, as compared to the situation prior to the health emergency caused by the COVID-19 pandemic. As a whole, these workers have a high SOC and are associated with WE in its three dimensions: Vigour, Absorption, and Dedication, as well as with PD, but inversely. Work environment is significantly correlated with the level of safety and effectiveness provided by the companies to protect themselves from infection by COVID-19 or the distance maintained by colleagues to avoid infection. The degree of job satisfaction during the pandemic or the perception of being appreciated as a healthcare professional by society are variables that condition the SOC, while the level of conflict in the workplace or the level of stress are inversely associated. Finally, it is worth noting the great importance given to the need for psychological support for those affected by the disease, the healthcare professionals involved in the treatment, and even the general population.

## Data Availability

Data are available online as Supplementary Material of the present article. Additional data and materials related to the study are available from the corresponding author.
