# Supplementary material for: Sense of Coherence in Healthcare Workers During the COVID-19 Pandemic in Ecuador: Association With Work Engagement, Work Environment and Psychological Distress Factors
Source: Int J Public Health. 2022 Dec 5;67:1605428. doi: 10.3389/ijph.2022.1605428 (PMC9760665; doi:10.3389/ijph.2022.1605428)
Supplement: Supplementary file 1 [file DataSheet1.docx]

**EIQ COVID-19 ECUADOR**

**Beginning of the questionnaire: Informed consent ***

Q1.1

INFORMED CONSENT OF THE PARTICIPANT

Name of the study: "Evaluation of the Impact of the COVID-19 Pandemic on the Emotional Well-being and Psychological Adjustment of Health Professionals and the General Population".

We are a team of researchers made up of professors and professionals from the Espírito Santo University of Specialties (Ecuador), the University of Huelva (Spain) and the Loyola Andalusia University (Spain), members of officially recognized research groups in areas such as Risk Prevention Labor, Psychology, Mental Health and Healthy Habits, and Research Methods and Techniques in Social and Health Sciences. We are conducting a study in Ecuador to learn about the psychological impact that the COVID-19 (coronavirus) pandemic is having on both the general population and health professionals. The information obtained will allow us to identify fundamental factors to ensure the best emotional well-being and psychological adjustment in an emergency such as the one we are currently experiencing. To do this, we need the participants to answer this questionnaire from which we will extract the data.

You must know that:

1) Your participation in this study is completely voluntary.

2) Participation or non-participation will not entail any benefit or harm to you.

3) All the data obtained in this study will be confidential and will be treated in accordance with Organic Law on Protection of Personal Data and Guarantees of Digital Rights 3/2018 of Spain and the Data Protection Legislation of Ecuador.

4) The information obtained will only be used for the specific purposes of the study.

5) Once you start answering the questionnaire, you are free to leave the study at any time, without assuming any consequences for you.

We provide you with the data of the contact researchers of this study:

• MSc. Cristian Arias Ulloa Director of the Master's Degree in Occupational Health and Safety. Espírito Santo University. Guayaquil. Ecuador e-mail: cariasu@uees.edu.ec

• Dr. Carlos Ruiz Frutos, MD, PhD. Professor of the Department of Sociology, Social Work and Public Health. University of Huelva. Spain. e-mail: fruits@uhu.es

• Dr. Juan Gómez Salgado, PhD. Professor of the Department of Sociology, Social Work and Public Health. University of Huelva. Spain. e-mail: saledo@uhu.es

• Dr. Sara Domínguez Salas, PhD. Professor of the Department of Psychology. Loyola Andalusia University. Spain. e-mail: sdominguez@uloyola.es

• Dr. Antonio R. Gómez García, PhD. Professor and Researcher of Safety and Health at Work. Espírito Santo University. Guayaquil. Ecuador e-mail: agomezg@uees.edu.ec

You declare that you (you must check both options):

are of legal age (18 or over) (1)

agree to participate in this study (2)

**Group 1: Sociodemographic data**

Q2.1 Sex*

- Man (1)
- Woman (2)

Q2.2 Age (enter a number): _________________

Q2.3 Marital status:

- Single (1)
- Married or living with a partner (2)
- Separated or Divorced (3)
- Widow/er (4)

Q2.4 Last completed studies:

- Without studies (1)
- Primary basic education (2)
- Secondary education (3)
- Technical - Technological (4)
- University (7)
- Master (8)
- PhD (9)

Q2.5 In which **province do you** live or are you currently undergoing the confinement decreed by the Government? (*select a province from the list*)

- Province (1)

▼ Azuay (1) ... Zamora Chinchipe (24)

Q2.6 In what **locality** (city, canton, parish, or enclosure) do you live or are you currently undergoing the confinement decreed by the Government? (*write the town*)______________________

Q2.7 What type of housing do you currently live in?

- Suit (1)
- Department (3)
- One Floor (Villa) (4)
- One Floor + 1 high floor (6)
- One Floor + 2 high floors (7)
- Others (indicate which one(s) (8) ____________________________________________

Q2.8 Do you have children? (*indicate how many)*

- Yes (enter a number) (1) _____________________________________________
- No (2)

Q2.9 Do you have a pet? (indicate which one(s) and quantity of each)

- Yes (indicate which one(s) and quantity of each) (1) _____________________________
- No (2)

Q2.10 Indicate your work situation (*you can select several options)*

- I am a student (1)
- I am currently working part-time FROM HOME (2)
- I am currently working part-time AWAY FROM HOME (3)
- I am currently working full time FROM HOME (4)
- I am currently working full time AWAY FROM HOME (5)
- I am unemployed (6)
- I am retired (7)
- I'm on a medical leave (8)
- Others (indicate which one(s)) (9) ____________________________________________

Show this question:

If Please indicate your employment status (you may select multiple options) = I am currently working part-time FROM HOME

Or Indicate your employment status (you can select several options) = I am currently working part-time AWAY FROM HOME

Or Indicate your employment status (you can select several options) = I am currently working full time FROM HOME

Or Indicate your employment status (you can select several options) = I am currently working full time AWAY FROM HOME

Q2.11 You are:

- Self-employed (1)
- Public employee (2)
- Worker of a private company (3)

Show this question:

If Please indicate your employment status (you may select multiple options) = I am currently working part-time FROM HOME

Or Indicate your employment situation (you can select several options) = I am currently working part-time AWAY FROM HOME

Or Indicate your employment status (you can select multiple options) = I am currently working full time FROM HOME

Or Indicate your employment situation (you can select several options) = I am currently working full time AWAY FROM HOME

Q2.12 Among the following occupations, please indicate which one best suits your job position:

Select an option (1)

▼ Farmers and skilled workers of agricultural exploitations destined for the market (1) ... ANOTHER OPTION NOT contemplated in the previous ones (44)

Show this question:

If Among the following occupations, please indicate which is the one that best suits your position d... = ANOTHER OPTION NOT contemplated in the previous ones

Q2.13 Write your occupation: _______________________________________

**Group 2: Health Professional**

Q3.1 Are you an active health services professional?

- Yes (1)
- No (2)

Show this question:

If Are you an active health services professional? = Yes

Q3.2 What is your occupation? (*you can mark several options)*

- Care (direct contact with the patient) (1)
- Researcher (2)
- Teacher (3)
- Management and/or Administrative (4)

Show this question:

If What is your occupation? (You can mark several options) = Care (direct contact with the patient)

And what is your occupation? (you can mark several options) = Researcher

And what is your occupation? (you can mark several options) = Teacher

And what is your occupation? (you can mark several options) = Management and/or Administrative

Q3.5 Could you indicate how many years of experience you have in each of the following fields?

|  | From 0 to 5 years (1) | From 5 to 10 years (2) | More than 10 years (3) |
| --- | --- | --- | --- |
| Care (1) |  |  |  |
| Research (2) |  |  |  |
| Management and/or Administrative (4) |  |  |  |

Show this question:

If What is your occupation? (you can mark several options) = Care (direct contact with the patient)

And what is your occupation? (you can mark several options) = Teacher

And what is your occupation? (you can mark several options) = Management and/or Administrative

And what is your occupation? (you can mark several options) = Researcher

Q3.6 Could you indicate how many years of experience you have in each of the following fields?

|  | From 0 to 5 years (1) | From 5 to 10 years (2) | More than 10 years (3) |
| --- | --- | --- | --- |
| Care (1) |  |  |  |
| Research (2) |  |  |  |
| Management and/or Administrative (4) |  |  |  |

Show this question:

If What is your occupation? (you can mark several options) = Researcher

And what is your occupation? (you can mark several options) = Teacher

And what is your occupation? (you can mark several options) = Management and/or Administrative

And what is your occupation? (you can mark several options) = Care (direct contact with the patient)

Q3.7 Could you indicate how many years of experience you have in each of the following fields?

|  | From 0 to 5 years (1) | From 5 to 10 years (2) | More than 10 years (3) |
| --- | --- | --- | --- |
| Research (1) |  |  |  |
| Teaching (2) |  |  |  |
| Management and/or Administrative (3) |  |  |  |

Show this question:

If What is your occupation? (you can mark several options) = Care (direct contact with the patient)

And what is your occupation? (you can mark several options) = Researcher

And what is your occupation? (you can mark several options) = Teacher

And what is your occupation? (you can mark several options) = Management and/or Administrative

Q3.8 Could you indicate how many years of experience you have in each of the following fields?

|  | From 0 to 5 years (1) | From 5 to 10 years (2) | More than 10 years (3) |
| --- | --- | --- | --- |
| Healthcare (1) |  |  |  |
| Research (2) |  |  |  |

Show this question:

If What is your occupation? (you can mark several options) = Care (direct contact with the patient)

And what is your occupation? (you can mark several options) = Teacher

And what is your occupation? (you can mark several options) != Researcher

And what is your occupation? (you can mark several options) != Management and/or Administrative

Q3.9 Could you indicate how many years of experience you have in each of the following areas?

|  | From 0 to 5 years (1) | From 5 to 10 years (2) | More than 10 years (3) |
| --- | --- | --- | --- |
| Healthcare (1) |  |  |  |
| Teaching (2) |  |  |  |

Show this question:

If What is your occupation? (you can mark several options) = Care (direct contact with the patient)

And what is your occupation? (you can mark several options) = Management and/or Administrative

And what is your occupation? (you can mark several options) = Researcher

And what is your occupation? (you can mark several options) = Teacher

Q3.10 Could you indicate how many years of experience you have in each of the following fields?

|  | From 0 to 5 years (1) | From 5 to 10 years (2) | More than 10 years (3) |
| --- | --- | --- | --- |
| Care (1) |  |  |  |
| Management and/or Administrative (2) |  |  |  |

Show this question:

If What is your occupation? (you can mark several options) = Researcher

And what is your occupation? (you can mark several options) = Management and/or Administrative

And what is your occupation? (you can mark several options) = Care (direct contact with the patient)

And what is your occupation? (you can mark several options) = Teacher

Q3.11 Could you indicate how many years of experience you have in each of the following areas?

|  | From 0 to 5 years (1) | From 5 to 10 years (2) | More than 10 years (3) |
| --- | --- | --- | --- |
| Research (1) |  |  |  |
| Management and/or Administrative (2) |  |  |  |

Show this question:

If What is your occupation? (you can mark several options) = Researcher

And what is your occupation? (you can mark several options) = Teacher

And what is your occupation? (you can mark several options) = Care (direct contact with the patient)

And what is your occupation? (you can mark several options) = Management and/or Administrative

Q3.12 Could you indicate how many years of experience you have in each of the following fields?

|  | From 0 to 5 years (1) | From 5 to 10 years (2) | More than 10 years (3) |
| --- | --- | --- | --- |
| Care (1) |  |  |  |
| Research (2) |  |  |  |

Show this question:

If What is your occupation? (You can mark several options) = Care (direct contact with the patient)

And what is your occupation? (you can mark several options) = Teacher

And what is your occupation? (you can mark several options) = Researcher

And what is your occupation? (you can mark several options) = Management and/or Administrative

Q3.13 Could you indicate how many years of experience you have in the following field?

|  | From 0 to 5 years (1) | From 5 to 10 years (2) | More than 10 years (3) |
| --- | --- | --- | --- |
| Care (1) |  |  |  |

Show this question:

If What is your occupation? (you can mark several options) = Care (direct contact with the patient)

And what is your occupation? (you can mark several options) = Teacher

And what is your occupation? (you can mark several options) = Researcher

And what is your occupation? (you can mark several options) = Management and/or Administrative

Q3.14 Could you indicate how many years of experience you have in the following field?

|  | From 0 to 5 years (1) | From 5 to 10 years (2) | More than 10 years (3) |
| --- | --- | --- | --- |
| Research (1) |  |  |  |

Show this question:

If What is your occupation? (you can mark several options) = Care (direct contact with the patient)

And what is your occupation? (you can mark several options) = Teacher

And what is your occupation? (you can mark several options) = Researcher

And what is your occupation? (you can mark several options) = Management and/or Administrative

Q3.15 Could you indicate how many years of experience you have in the following field?

|  | From 0 to 5 years (1) | From 5 to 10 years (2) | More than 10 years (3) |
| --- | --- | --- | --- |
| Teaching (1) |  |  |  |

Show this question:

If What is your occupation? (you can mark several options) = Care (direct contact with the patient)

And what is your occupation? (you can mark several options) = Teacher

And what is your occupation? (you can mark several options) = Researcher

And what is your occupation? (you can mark several options) = Management and/or Administrative

Q3.16 Could you indicate how many years of experience you have in the following field?

|  | From 0 to 5 years (1) | From 5 to 10 years (2) | More than 10 years (3) |
| --- | --- | --- | --- |
| Management and/or Administrative (1) |  |  |  |

Show this question:

If Are you an active health services professional? = Yes

Q3.17 Indicate your active professional profile:

Select an option (1)

▼ Nursing assistants (1) ... ANOTHER OPTION not considered among the above (86)

Show this question:

If Indicate your active professional profile: = ANOTHER OPTION not considered among the above

Q3.18 Write your active professional profile: __________________________________________

Show this question:

If What is your occupation? (You can mark several options) = Care (direct contact with the patient)

Or What is your occupation? (you can mark several options) = Researcher

Or What is your occupation? (you can mark several options) = Teacher

Or What is your occupation? (you can mark several options) = Management and/or Administrative

Q3.19 You currently work in a center which is: *(you can mark several options)*

- Public (1)
- Private (2)
- Public/private attention (3)
- Others (indicate which one/s) (5) _____________________________________________

Show this question:

If What is your occupation? (You can mark several options) = Care (direct contact with the patient)

Or What is your occupation? (you can mark several options) = Management and/or Administrative

Q3.20 You work in:

- Primary care (non-hospital) (1)
- Hospital care (general and specialist hospitals) (2)
- Others (indicate which one/s) (3) _____________________________________________

Show this question:

If What is your occupation? (you can mark several options) = Care (direct contact with the patient)

And what is your occupation? (you can mark several options) = Researcher

And what is your occupation? (you can mark several options) = Teacher

And what is your occupation? (you can mark several options) = Management and/or Administrative

Q3.21 Do you feel capable of facing your imminent incorporation into the care setting given the current situation?

- Yes (1)
- No (2)
- I don't know (3)

Show this question:

If What is your occupation? (you can mark several options) = Care (direct contact with the patient)

And what is your occupation? (you can mark several options) = Researcher

And what is your occupation? (you can mark several options) = Teacher

And what is your occupation? (you can mark several options) = Management and/or Administrative

Q3.22 Do you feel capable of facing your incorporation into the healthcare field imminently given the current situation?

- Yes (1)
- No (2)
- I don't know (3)

Show this question:

If What is your occupation? (you can mark several options) = Care (direct contact with the patient)

And what is your occupation? (you can mark several options) = Researcher

And what is your occupation? (you can mark several options) = Teacher

And what is your occupation? (you can mark several options) = Management and/or Administrative

Q3.23 Do you feel capable of facing your incorporation into the healthcare field imminently given the current situation?

- Yes (1)
- No (2)
- I don't know (3)

Show this question:

If What is your occupation? (you can mark several options) = Care (direct contact with the patient)

And what is your occupation? (you can mark several options) = Researcher

And what is your occupation? (you can mark several options) = Teacher

And what is your occupation? (you can mark several options) = Management and/or Administrative

Q3.24 Do you feel capable of facing your incorporation into the healthcare field imminently given the current situation?

- Yes (1)
- No (2)
- I don't know (3)

Show this question:

If What is your occupation? (you can mark several options) = Care (direct contact with the patient)

And what is your occupation? (you can mark several options) = Researcher

And what is your occupation? (you can mark several options) = Teacher

And what is your occupation? (you can mark several options) = Management and/or Administrative

Q3.25 Do you feel capable of facing your incorporation into the healthcare field imminently given the current situation?

- Yes (1)
- No (2)
- I don't know (3)

Show this question:

If What is your occupation? (you can mark several options) = Care (direct contact with the patient)

And what is your occupation? (you can mark several options) = Researcher

And what is your occupation? (you can mark several options) = Teacher

And what is your occupation? (you can mark several options) = Management and/or Administrative

Q3.26 Do you feel capable of facing your imminent incorporation into the care setting given the current situation?

- yes (1)
- No (2)
- I don't know (3)

**Group 3: Available Information on COVID-19**

Q4.1 Through what means or platforms has the information about COVID-19 reached you or is it reaching you? (*you can select several options*)

- Social Networks (WhatsApp, Facebook, Instagram, etc.) (1)
- Radio (2)
- Google or other search engines (9)
- Web pages of **official organizations** or scientific societies (3)
- Telephones or **official information Apps** (4)
- Television (5)
- Newspapers (online or paper) (6)
- Friends or family (7)
- Others (indicate which one/s) (8) _____________________________________________

Q4.2 How many hours a day do you estimate that you watch, read or listen to news related to COVID-19?

Select an option (1)

▼ 0 (1) ... more than 20 (22)

Q4.3 Evaluate how you consider that the information provided by the **media** about COVID-19 is being treated in terms of...

|  | Very low (1) | Low (2) | Medium (3) | High (4) | Very high (5) |
| --- | --- | --- | --- | --- | --- |
| Accessibility (comes to you)(1) |  |  |  |  |  |
| Quantity (2) |  |  |  |  |  |
| Quality (3) |  |  |  |  |  |
| Utility (4) |  |  |  |  |  |

Q4.4 Evaluate how you consider the information provided by **official channels** (government entities) about COVID-19 is being treated in terms of...

|  | Very low (1) | Low (2) | Medium (3) | High (4) | very high (5) |
| --- | --- | --- | --- | --- | --- |
| Accessibility (comes to you)(1) |  |  |  |  |  |
| Quantity (2) |  |  |  |  |  |
| Quality (3) |  |  |  |  |  |
| Utility (4) |  |  |  |  |  |

Q4.5 Do you check the veracity of the information you receive through other official sources?

- Yes (1)
- No (2)

Q4.6 Do you think you have enough information regarding COVID-19 about...

|  | 1-Not at all agree (1) | 2 (2) | 3 (3) | 4 (4) | 5 (5) | 6 (6) | 7 (7) | 8 (8) | 9 (9) | 10-Strongly agree (10) |
| --- | --- | --- | --- | --- | --- | --- | --- | --- | --- | --- |
| Its symptoms (1) |  |  |  |  |  |  |  |  |  |  |
| Its prognosis (2) |  |  |  |  |  |  |  |  |  |  |
| Its treatment (3) |  |  |  |  |  |  |  |  |  |  |
| Its transmission routes (4) |  |  |  |  |  |  |  |  |  |  |
| Its preventive measures (5) |  |  |  |  |  |  |  |  |  |  |

Show this question:

If Please, indicate your employment status (you may select multiple options) = I am currently working part-time FROM HOME

Or Indicate your employment situation (you can select several options) = I am currently working part-time AWAY FROM HOME

Or Indicate your employment status (you can select multiple options) = I am currently working full time FROM HOME

Or Indicate your employment situation (you can select several options) = I am currently working full time AWAY FROM HOME

Q4.7 Do you believe that your department, service, unit or company has provided you with clear (and accurate) information about the COVID-19 pandemic?

- I don't agree (1)
- 2 (2)
- 3 (3)
- 4 (4)
- 5 (5)
- 6 (6)
- 7 (7)
- 8 (8)
- 9 (9)
- 10 - Totally agree (10)

**Group 4: Prevention measures**

Q5.1 Please indicate how often you have performed these preventive measures in the last 14 days.
Covering your mouth using your elbow when you cough or sneeze:

- Never (1)
- Rarely (6)
- Sometimes (7)
- Almost always (8)
- Always (9)

Q5.2 Avoiding sharing utensils (eg. fork) during meals:

- Never (1)
- Rarely (2)
- Sometimes (3)
- Almost always (4)
- Always (5)

Q5.3 Washing hands with soap and water:

- Never (1)
- Rarely (2)
- Sometimes (3)
- Almost always (4)
- Always (5)

Q5.4 Washing hands with alcohol-based hand sanitizer:

- Never (1)
- Rarely (2)
- Sometimes (3)
- Almost always (4)
- Always (5)

Q5.5 Washing hands immediately after coughing, touching your nose, or sneezing:

- Never (1)
- Rarely (2)
- Sometimes (3)
- Almost always (4)
- Always (5)

Q5.6 Washing hands after touching potentially contaminated objects:

- Never (1)
- Rarely (2)
- Sometimes (3)
- Almost always (4)
- Always (5)

Q5.7 Wearing a mask regardless of the presence or absence of symptoms:

- Never (1)
- Rarely (2)
- Sometimes (3)
- Almost always (4)
- Always (5)

Q5.8 Leaving at least a meter and a half distance from others:

- Never (1)
- Rarely (2)
- Sometimes (3)
- Almost always (4)
- Always (5)

Q5.9 How effective do you think preventive measures can be?

- 1-Not at all effective (1)
- 2 (2)
- 3 (3)
- 4 (4)
- 5 (5)
- 6 (6)
- 7 (7)
- 8 (8)
- 9 (9)
- 10 - Very effective (10)

Q5.10 Are you complying with the confinement decreed by the Government?

- Yes, in strict confinement (1)
- Yes, in confinement, going out to buy and/or work (2)
- I do not comply with confinement (3)
- Other situations (indicate which one/s) (4) ____________________________________

Show this question:

If Are you complying with the confinement decreed by the Government? = I do not comply with confinement

Q5.11 How many days have you been confined or reducing contact with others?

No. of days (1)

▼ 0 (1) ... more than 60 days (19)

Q5.12 How many people do you live with (NOT including yourself) in the confinement decreed by the Government?

No. of people (1)

▼ 0 (1) ... more than 20 (22)

Show this question:

If How many people do you live with (NOT including yourself) in the confinement decreed by the Government? = 0

Q5.13 How many of them are people at risk*?

** Older adults, pregnant women, and people with serious chronic medical conditions (diabetes, heart disease, heart and/or lung disease, immunocompromised).*

Number of people at risk (1)

▼ 0 (1) ... more than 20 (22)

Show this question:

If How many people do you live with (NOT including yourself) in the confinement decreed by the Government? = 0

Q5.14 How many of the people you live with are children or adolescents?

Select a number (1)

▼ 0 (1) ... more than 20 (22)

Show this question:

Marital Status: = Separated or Divorced

And do you have children? (indicate how many) = Yes (enter a number)

Q5.15 Briefly describe the joint custody that you are currently maintaining concerning your children._______________________________________________________________________

Show this question:

If How many people do you live with (NOT including yourself) in the confinement decreed by the Government? = 0

Q5.16 How **many** of the people you live with have physical and/or intellectual disabilities?

- None (4)
- Physical disability (insert a number) (1) ________________________________________
- Intellectual disability (insert a number) (2) ________________________________
- Other type of disability (indicate which one/s and number of people) (3) _______________

Show this question:

If Indicate your employment situation (you can select several options) = I am currently working part-time AWAY FROM HOME

Or Indicate your employment situation (you can select several options) = I am currently working full time AWAY FROM HOME

Q5.17 Do you perform the recommended preventive measures...

|  | Yes (1) | No (2) |
| --- | --- | --- |
| Inside the workplace? (1) |  |  |
| Outside the workplace? (2) |  |  |

Show this question:

If Please, indicate your employment status (you can select multiple options) = I am currently working part-time FROM HOME

Or Indicate your employment situation (you can select several options) = I am currently working part-time AWAY FROM HOME

Or Indicate your employment status (you can select multiple options) = I am currently working full time FROM HOME

Or Indicate your employment situation (you can select several options) = I am currently working full time AWAY FROM HOME

Q5.18 Have you received specific information for the prevention of COVID-19 infection from your department, service, unit or company?

- Yes (1)
- No (2)

**Group 5: Beliefs about the outbreak**

Q6.1 How would you rate your concern about COVID-19?

- Not worried (1)
- 2 (2)
- 3 (3)
- 4 (4)
- 5 (5)
- 6 (6)
- 7 (7)
- 8 (8)
- 9 (9)
- 10 - Very worried (10)

Q6.2 How likely do you think you are to survive COVID-19 if you are or become infected?

- 1 - Not likely (1)
- 2 (2)
- 3 (3)
- 4 (4)
- 5 (5)
- 6 (6)
- 7 (7)
- 8 (8)
- 9 (9)
- 10- Very likely (10)

Q6.3 What level of confidence do you have in the ability of **health professionals** to diagnose or recognize COVID-19?

- 1 - No confidence (1)
- 2 (2)
- 3 (3)
- 4 (4)
- 5 (5)
- 6 (6)
- 7 (7)
- 8 (8)
- 9 (9)
- 10- Total confidence (10)

Q6.4 What level of confidence do you have in the ability of **the health system** to diagnose or recognize COVID-19?

- 1 - No confidence (1)
- 2 (2)
- 3 (3)
- 4 (4)
- 5 (5)
- 6 (6)
- 7 (7)
- 8 (8)
- 9 (9)
- 10- Total confidence (10)

Q6.5 What do you think is the risk of your getting infected by COVID-19?

- 1 - Very low (1)
- 2 (2)
- 3 (3)
- 4 (4)
- 5 (5)
- 6 (6)
- 7 (7)
- 8 (8)
- 9 (9)
- 10- Very high (10)

Q6.6 Do you think that getting infected with COVID-19 would have serious consequences for your health?

- 1 - No consequence (1)
- 2 (2)
- 3 (3)
- 4 (4)
- 5 (5)
- 6 (6)
- 7 (7)
- 8 (8)
- 9 (9)
- 10- Many consequences (10)

Q6.7 Do you think the infection is difficult to treat?

- 1 - Not difficult at all (1)
- 2 (2)
- 3 (3)
- 4 (4)
- 5 (5)
- 6 (6)
- 7 (7)
- 8 (8)
- 9 (9)
- 10- Very difficult (10)

Q6.8 To what extent are you worried about getting infected with COVID-19?

- 1 - I am not worried at all (1)
- 2 (2)
- 3 (3)
- 4 (4)
- 5 (5)
- 6 (6)
- 7 (7)
- 8 (8)
- 9 (9)
- 10- I am very worried (10)

Q6.9 To what extent are you worried about being a carrier and transmitting the virus to family members, close people or patients?

- 1 - I am not worried at all (1)
- 2 (2)
- 3 (3)
- 4 (4)
- 5 (5)
- 6 (6)
- 7 (7)
- 8 (8)
- 9 (9)
- 10- I am very worried (10)

**Group 6: Symptomatology**

Q7.1 How have you perceived your health in the last two weeks?

- Lousy (1)
- Bad (2)
- Mediocre (3)
- Good (4)
- Very good (5)

Q7.2 Do you suffer from any type of disability?

- Yes (indicate which one(s)) (1) ________________________________________________
- No (2)

Q7.3 In the last two weeks, have you had...

|  | Yes (1) | No (2) |
| --- | --- | --- |
| Fever (at least one day with 38ºC or more) (1) |  |  |
| Cough (2) |  |  |
| Headache (3) |  |  |
| Muscle pain (4) |  |  |
| Dizziness (5) |  |  |
| Diarrhea (6) |  |  |
| Sore throat (7) |  |  |
| Coryza (8) |  |  |
| Chills (9) |  |  |
| Breathing difficulties (10) |  |  |

Q7.4 Do you suffer from any of the following chronic diseases? (*you can select several options)*:

- I do not suffer from any chronic disease (13)
- Active cancer (4)
- Diabetes (5)
- Immunosuppressive disease or situation (6)
- Chronic cardiovascular disease (*ischemic heart disease, joint fibrillation, heart failure*) (7)
- Chronic respiratory disease (*asthma, bronchiectasis, COPD, pulmonary fibrosis*) (8)
- High blood pressure (9)
- Metabolic syndrome (10)
- I suffer from other chronic diseases (indicate which one/s) (11) ______________________

Q7.5 Are you currently taking any type of medication?

- Yes (indicate which one/s) (1) _______________________________________________
- No (2)

Q7.6 Have you had a recent hospitalization in the past 14 days?

- Yes (1)
- No (2)

Q7.7 Have you received medical attention in a health center, hospital or clinic in the last 14 days?

- Yes (1)
- No (2)

**Group 7: Direct contact and diagnosis**

Q8.1 Have you been in close contact (contact for **more than 15 minutes at less than two meters)** with a confirmed infected person?

- Yes (1)
- No (5)
- Probably yes (2)
- Probably no (3)
- I don't know (4)

Q8.2 Have you been in casual contact (all other contacts) with a confirmed infected person?

- Yes (1)
- No (2)
- Probably yes (3)
- Probably no (4)
- I don't know (5)

Q8.3 Have you had any type of contact with any person or material suspected of being infected?

- Yes (1)
- No (2)
- Probably yes (3)
- Probably no (4)
- I don't know (5)

Q8.4 Has any member of your family been infected with COVID-19?

- Yes (1)
- No (2)
- Probably yes (3)
- Probably no (4)
- I don't know (5)

Show this question:

If Has any member of your family been infected with COVID-19? = Yes

Or Has any member of your family been infected with COVID-19? = Probably yes

Q8.5 Do you live with this family member?

- Yes (1)
- No (2)
- Other situation (indicate which one(s)) (3) _______________________________________

Show this question:

If Please, indicate your employment status (you may select multiple options) = I am currently working part-time FROM HOME

Or Indicate your employment status (you can select several options) = I am currently working part-time AWAY FROM HOME

Or Indicate your employment status (you can select several options) = I am currently working full time FROM HOME

Or Indicate your employment situation (you can select several options) = I am currently working full time AWAY FROM HOME

Q8.6 Has a co-worker been infected by COVID-19?

- Yes (1)
- No (2)
- Probably yes (3)
- Probably no (4)
- I don't know (5)

Q8.7 Are you in quarantine due to presenting symptoms or having had any risk contact?

- Yes (1)
- No (2)
- Other situation (indicate which one/s) (3) __________________________________________

Q8.8 Have you had a diagnostic test for COVID-19?

- Yes (1)
- No (2)

Show this question:

If Have you had a diagnostic test for COVID-19? = yes

Q8.9 On what date? (*indicate if possible the day and month)* __________________________________

Show this question:

If Have you had a diagnostic test for COVID-19? = yes

Q8.10 The result of the test has been:

- Positive (1)
- Negative (2)
- I don't know (3)

Show this question:

If Have you had a diagnostic test for COVID-19? = no

Or The test result was: = I don't know

Q8.11 Do you consider that you may have contracted the COVID-19 virus?

- Yes (1)
- No (2)
- Probably yes (3)
- Probably no (4)
- I don't know (5)

**Group 8: Work environment**

Show this question:

If Please, indicate your employment status (you can select several options) = I am currently working part-time AWAY FROM HOME

Or Indicate your employment status (you can select several options) = I am currently working full time AWAY FROM HOME

Or What is your occupation? (you can mark several options) = Care (direct contact with the patient)

Or What is your occupation? (you can mark several options) = Management and/or Administrative

Q9.1 Have you restricted your contact with friends and/or relatives because you consider your work environment to be dangerous?

- Yes (1)
- No (2)

Show this question:

If Indicate your employment situation (you can select several options) = I am currently working part-time AWAY FROM HOME

Or Indicate your employment situation (you can select several options) = I am currently working full time AWAY FROM HOME

Or What is your occupation? (You can mark several options) = Care (direct contact with the patient)

Or What is your occupation? (you can mark several options) = Management and/or Administrative

Q9.2 Do you think that your family and/or friends avoid you because you work in an environment with a high risk of infection?

- Yes (1)
- No (2)
- I don't know (3)

Show this question:

If Please, indicate your employment status (you can select multiple options) = I am currently working part-time FROM HOME

Or Indicate your employment status (you can select several options) = I am currently working part-time AWAY FROM HOME

Or Indicate your employment status (you can select several options) = I am currently working full time FROM HOME

Or Indicate your employment status (you can select several options) = I am currently working full time AWAY FROM HOME

Q9.3 Do you think that your department, service, unit or company has provided you with the necessary material and means to carry out your work EFFECTIVELY?

- Absolutely no (1)
- 2 (2)
- 3 (3)
- 4 (4)
- 5 (5)
- 6 (6)
- 7 (7)
- 8 (8)
- 9 (9)
- 10 - Absolutely yes (10)

Show this question:

If Please, indicate your employment status (you may select multiple options) = I am currently working part-time FROM HOME

Or Indicate your employment status (you can select several options) = I am currently working part-time AWAY FROM HOME

Or Indicate your employment status (you can select several options) = I am currently working full time FROM HOME

Or Indicate your employment status (you can select several options) = I am currently working full time AWAY FROM HOME

Q9.4 Do you think that your department, service, unit or company has provided you with the necessary material and means to carry out your work SAFELY?

- Absolutely no (1)
- 2 (2)
- 3 (3)
- 4 (4)
- 5 (5)
- 6 (6)
- 7 (7)
- 8 (8)
- 9 (9)
- 10 - Absolutely yes (10)

Show this question:

If Please, indicate your employment status (you may select multiple options) = I am currently working part-time FROM HOME

Or Indicate your employment status (you can select several options) = I am currently working part-time AWAY FROM HOME

Or Indicate your employment status (you can select several options) = I am currently working full time FROM HOME

Or Indicate your employment status (you can select several options) = I am currently working full time AWAY FROM HOME

Q9.5 What material and means are missing in your department, service, unit or company? (*indicate what you consider below)*

________________________________________________________________________________________________________________________________________________________________________________________________________________________________________________________________________________________________________________________

Show this question:

If Please, indicate your employment status (you can select several options) = I am currently working part-time AWAY FROM HOME

Or Indicate your employment status (you can select several options) = I am currently working full time AWAY FROM HOME

Q9.6 Do you think that the separation distance maintained with your colleagues is adequate?

- Absolutely no (1)
- 2 (2)
- 3 (3)
- 4 (4)
- 5 (5)
- 6 (6)
- 7 (7)
- 8 (8)
- 9 (9)
- 10 - Absolutely yes (10)

Show this question:

If Please, indicate your employment status (you can select several options) = I am currently working part-time AWAY FROM HOME

Or Indicate your employment status (you can select several options) = I am currently working full time AWAY FROM HOME

Q9.7 Are you in contact with clients/users/patients who could be a source of risk?

- Absolutely no (1)
- 2 (2)
- 3 (3)
- 4 (4)
- 5 (5)
- 6 (6)
- 7 (7)
- 8 (8)
- 9 (9)
- 10 - Absolutely yes (10)

Show this question:

If Please, indicate your employment status (you can select multiple options) = I am currently working part-time FROM HOME

Or Indicate your employment situation (you can select several options) = I am currently working part-time AWAY FROM HOME

Or Indicate your employment status (you can select multiple options) = I am currently working full time FROM HOME

Or Indicate your employment status (you can select several options) = I am currently working full time AWAY FROM HOME

Q9.8 Have you observed an increase in labour conflict in your work?

- Absolutely no (1)
- 2 (2)
- 3 (3)
- 4 (4)
- 5 (5)
- 6 (6)
- 7 (7)
- 8 (8)
- 9 (9)
- 10 - Absolutely yes (10)

Show this question:

If Please, indicate your employment status (you may select multiple options) = I am currently working part-time FROM HOME

Or Indicate your employment status (you can select several options) = I am currently working part-time AWAY FROM HOME

Or Indicate your employment status (you can select several options) = I am currently working full time FROM HOME

Or Indicate your employment status (you can select several options) = I am currently working full time AWAY FROM HOME

Q9.9 Do you think that your profession or your workplace puts you at risk of being infected?

- Absolutely no (1)
- 2 (2)
- 3 (3)
- 4 (4)
- 5 (5)
- 6 (6)
- 7 (7)
- 8 (8)
- 9 (9)
- 10 - Absolutely yes (10)

Show this question:

If Please, indicate your employment status (you may select multiple options) = I am currently working part-time FROM HOME

Or Indicate your employment situation (you can select several options) = I am currently working part-time AWAY FROM HOME

Or Indicate your employment status (you can select multiple options) = I am currently working full time FROM HOME

Or Indicate your employment situation (you can select several options) = I am currently working full time AWAY FROM HOME

Q9.10 Do you accept the risk of getting infected as part of your job?

- Absolutely no (1)
- 2 (2)
- 3 (3)
- 4 (4)
- 5 (5)
- 6 (6)
- 7 (7)
- 8 (8)
- 9 (9)
- 10 - Absolutely yes (10)

Q9.11 Do you think it would be important to offer a psychological support service to **professionals and volunteers** who are directly intervening in the COVID-19 health emergency?

- Absolutely no (1)
- 2 (2)
- 3 (3)
- 4 (4)
- 5 (5)
- 6 (6)
- 7 (7)
- 8 (8)
- 9 (9)
- 10 - Absolutely yes (10)

Q9.12 Do you think it would be important to offer a psychological support service to **individuals and families directly affected** by COVID-19 to face the difficulties generated by the health emergency?

- Absolutely no (1)
- 2 (2)
- 3 (3)
- 4 (4)
- 5 (5)
- 6 (6)
- 7 (7)
- 8 (8)
- 9 (9)
- 10 - Absolutely yes (10)

Q9.13 Do you think it would be important to offer a psychological support service to the **general population** to face the difficulties generated by the COVID-19 health emergency?

- Absolutely no (1)
- 2 (2)
- 3 (3)
- 4 (4)
- 5 (5)
- 6 (6)
- 7 (7)
- 8 (8)
- 9 (9)
- 10 - Absolutely yes (10)

Show this question:

If Please, indicate your employment status (you can select multiple options) = I am currently working part-time FROM HOME

Or Indicate your employment situation (you can select several options) = I am currently working part-time AWAY FROM HOME

Or Indicate your employment status (you can select multiple options) = I am currently working full time FROM HOME

Or Indicate your employment situation (you can select several options) = I am currently working full time AWAY FROM HOME

Q9.14 Do you consider that there has been an increase in your workload since the start of the health emergency?

- Absolutely no (1)
- 2 (2)
- 3 (3)
- 4 (4)
- 5 (5)
- 6 (6)
- 7 (7)
- 8 (8)
- 9 (9)
- 10 - Absolutely yes (10)

Show this question:

If Please, indicate your employment status (you may select multiple options) = I am currently working part-time FROM HOME

Or Indicate your employment status (you can select several options) = I am currently working part-time AWAY FROM HOME

Or Indicate your employment status (you can select several options) = I am currently working full time FROM HOME

Or Indicate your employment status (you can select several options) = I am currently working full time AWAY FROM HOME

Q9.15 Do you feel more stressed at work?

- Absolutely no (1)
- 2 (2)
- 3 (3)
- 4 (4)
- 5 (5)
- 6 (6)
- 7 (7)
- 8 (8)
- 9 (9)
- 10 - Absolutely yes (10)

Show this question:

If Please, indicate your employment status (you may select multiple options) = I am currently working part-time FROM HOME

Or Indicate your employment status (you can select several options) = I am currently working part-time AWAY FROM HOME

Or Indicate your employment status (you can select several options) = I am currently working full time FROM HOME

Or Indicate your employment status (you can select several options) = I am currently working full time AWAY FROM HOME

Q9.16 How would you rate the degree of satisfaction with your job in the current situation of COVID-19?

- Very dissatisfied (1)
- 2 (2)
- 3 (3)
- 4 (4)
- 5 (5)
- 6 (6)
- 7 (7)
- 8 (8)
- 9 (9)
- 10 - Totally satisfied (10)

Show this question:

If What is your occupation? (you can mark several options) = Care (direct contact with the patient)

Or What is your occupation? (you can mark several options) = Management and/or Administrative

Q9.17 As a health professional, do you feel appreciated by society?

- Absolutely no (1)
- 2 (2)
- 3 (3)
- 4 (4)
- 5 (5)
- 6 (6)
- 7 (7)
- 8 (8)
- 9 (9)
- 10 - Absolutely yes (10)

**Group 9: Work Scales**

Q10.1 Next, indicate YOUR PERCEPTION of certain variables related to your life and health habits, before the health emergency and at present.
To do so, score yourself from 1 to 10, with 1 being the least favorable situation and 10 the best.

The **quality of health care BEFORE** the health emergency:

Select a number (1)

▼ 1 (1) ... 10 (10)

Q10.2 **CURRENT quality of health care**:

Select a number (1)

▼ 1 (1) ... 10 (10)

Show this question:

If Please, indicate your employment status (you may select multiple options) = I am currently working part-time FROM HOME

Or Indicate your employment status (you can select several options) = I am currently working part-time AWAY FROM HOME

Or Indicate your employment status (you can select several options) = I am currently working full time FROM HOME

Or Indicate your employment status (you can select several options) = I am currently working full time AWAY FROM HOME

Q10.3 Working **conditions BEFORE** the health emergency:

Select a number (1)

▼ 1 (1) ... 10 (10)

Show this question:

If Please, indicate your employment status (you may select multiple options) = I am currently working part-time FROM HOME

Or Indicate your employment status (you can select several options) = I am currently working part-time AWAY FROM HOME

Or Indicate your employment status (you can select several options) = I am currently working full time FROM HOME

Or Indicate your employment status (you can select several options) = I am currently working full time AWAY FROM HOME

Q10.4 **CURRENT** **working conditions:**

Select a number (1)

▼ 1 (1) ... 10 (10)

Show this question:

If Please, indicate your employment status (you may select multiple options) = I am currently working part-time FROM HOME

Or Indicate your employment status (you can select several options) = I am currently working part-time AWAY FROM HOME

Or Indicate your employment status (you can select several options) = I am currently working full time FROM HOME

Or Indicate your employment status (you can select several options) = I am currently working full time AWAY FROM HOME

Q10.5 **Occupational health BEFORE** the health emergency:

Select a number (1)

▼ 1 (1) ... 10 (10)

Show this question:

If Please, indicate your employment status (you may select multiple options) = I am currently working part-time FROM HOME

Or Indicate your employment status (you can select several options) = I am currently working part-time AWAY FROM HOME

Or Indicate your employment status (you can select several options) = I am currently working full time FROM HOME

Or Indicate your employment status (you can select several options) = I am currently working full time AWAY FROM HOME

Q10.6 **CURRENT** **occupational health:**

Select a number (1)

▼ 1 (1) ... 10 (10)

Show this question:

If What is your occupation? (you can mark several options) = Care (direct contact with the patient)

Or What is your occupation? (you can mark several options) = Management and/or Administrative

Q10.7 **Patient safety BEFORE** the health emergency:

Select a number (1)

▼ 1 (1) ... 10 (10)

Show this question:

If What is your occupation? (you can mark several options) = Care (direct contact with the patient)

Or What is your occupation? (you can mark several options) = Management and/or Administrative

Q10.8 **CURRENT Patient safety**

Select a number (1)

▼ 1 (1) ... 10 (10)

Show this question:

If Please, indicate your employment status (you may select multiple options) = I am currently working part-time FROM HOME

Or Indicate your employment status (you can select several options) = I am currently working part-time AWAY FROM HOME

Or Indicate your employment status (you can select several options) = I am currently working full time FROM HOME

Or Indicate your employment status (you can select several options) = I am currently working full time AWAY FROM HOME

Q10.9 The following questions refer to people's feelings at work.
Please, read each question carefully and decide if you have felt this way in **the last week.**     
In my work, I feel full of energy:

- Never (1)
- Rarely (2)
- Sometimes (3)
- Regularly (4)
- Quite often (5)
- Almost always (6)
- Always (7)

Show this question:

If Please, indicate your employment status (you can select multiple options) = I am currently working part-time FROM HOME

Or Indicate your employment situation (you can select several options) = I am currently working part-time AWAY FROM HOME

Or Indicate your employment status (you can select multiple options) = I am currently working full time FROM HOME

Or Indicate your employment situation (you can select several options) = I am currently working full time AWAY FROM HOME

Q10.10 I am strong and vigorous in my work:

- Never (1)
- Rarely (2)
- Sometimes (3)
- Regularly (4)
- Quite often (5)
- Almost always (6)
- Always (7)

Show this question:

If Please, indicate your employment status (you may select multiple options) = I am currently working part-time FROM HOME

Or Indicate your employment status (you can select several options) = I am currently working part-time AWAY FROM HOME

Or Indicate your employment status (you can select several options) = I am currently working full time FROM HOME

Or Indicate your employment status (you can select several options) = I am currently working full time AWAY FROM HOME

Q10.11 I am enthusiastic about my work:

- Never (1)
- Rarely (2)
- Sometimes (3)
- Regularly (4)
- Quite often (5)
- Almost always (6)
- Always (7)

Show this question:

If Please, indicate your employment status (you can select multiple options) = I am currently working part-time FROM HOME

Or Indicate your employment situation (you can select several options) = I am currently working part-time AWAY FROM HOME

Or Indicate your employment status (you can select multiple options) = I am currently working full time FROM HOME

Or Indicate your employment status (you can select several options) = I am currently working full time AWAY FROM HOME

Q10.12 My work inspires me:

- Never (1)
- Rarely (2)
- Sometimes (3)
- Regularly (4)
- Quite often (5)
- Almost always (6)
- Always (7)

Show this question:

If Please, indicate your employment status (you may select multiple options) = I am currently working part-time FROM HOME

Or Indicate your employment status (you can select several options) = I am currently working part-time AWAY FROM HOME

Or Indicate your employment status (you can select multiple options) = I am currently working full time FROM HOME

Or Indicate your employment situation (you can select several options) = I am currently working full time AWAY FROM HOME

Q10.13 When I get up in the morning, I feel like going to work:

- Never (1)
- Rarely (2)
- Sometimes (3)
- Regularly (4)
- Quite often (5)
- Almost always (6)
- Always (7)

Show this question:

If Please, indicate your employment status (you can select multiple options) = I am currently working part-time FROM HOME

Or Indicate your employment status (you can select several options) = I am currently working part-time AWAY FROM HOME

Or Indicate your employment status (you can select several options) = I am currently working full time FROM HOME

Or Indicate your employment status (you can select several options) = I am currently working full time AWAY FROM HOME

Q10.14 I am happy when I am absorbed in my work:

- Never (1)
- Rarely (2)
- Sometimes (3)
- Regularly (4)
- Quite often (5)
- Almost always (6)
- Always (7)

Show this question:

If Please, indicate your employment status (you may select multiple options) = I am currently working part-time FROM HOME

Or Indicate your employment status (you can select several options) = I am currently working part-time AWAY FROM HOME

Or Indicate your employment status (you can select several options) = I am currently working full time FROM HOME

Or Indicate your employment status (you can select several options) = I am currently working full time AWAY FROM HOME

Q10.15 I am proud of the work I do:

- Never (1)
- Rarely (2)
- Sometimes (3)
- Regularly (4)
- Quite often (5)
- Almost always (6)
- Always (7)

Show this question:

If Please, indicate your employment status (you can select multiple options) = I am currently working part-time FROM HOME

Or Indicate your employment situation (you can select several options) = I am currently working part-time AWAY FROM HOME

Or Indicate your employment status (you can select multiple options) = I am currently working full time FROM HOME

Or Indicate your employment situation (you can select several options) = I am currently working full time AWAY FROM HOME

Q10.16 I am immersed in my work:

- Never (1)
- Rarely (2)
- Sometimes (3)
- Regularly (4)
- Quite often (5)
- Almost always (6)
- Always (7)

Show this question:

If Please, indicate your employment status (you can select multiple options) = I am currently working part-time FROM HOME

Or Indicate your employment situation (you can select several options) = I am currently working part-time AWAY FROM HOME

Or Indicate your employment status (you can select multiple options) = I am currently working full time FROM HOME

Or Indicate your employment situation (you can select several options) = I am currently working full time AWAY FROM HOME

Q10.17 I let myself be ‘carried away’ by my work (time flies when I am working):

- Never (1)
- Rarely (2)
- Sometimes (3)
- Regularly (4)
- Quite often (5)
- Almost always (6)
- Always (7)

**Group 10: Psychological Adjustment**

Q11.1 Next, we would like to know if you have any medical problems and how your health has been, in general, during the last few weeks.

Answer all the questions indicating the answer that, in your opinion, best applies to you.
Remember that you should only answer for recent (past two weeks) problems and problems you have now, not problems you had in the past.

Q11.1. Have you been able to concentrate well on what you were doing?

- Better than usual (1)
- Same as usual (2)
- Less than usual (3)
- Much less than usual (4)

Q11.2. Have your worries made you lose a lot of sleep?

- Not at all (1)
- No more than usual (2)
- Somewhat more than usual (3)
- Much more than usual (4)

Q11.3 Have you felt that you are playing a useful role in life?

- More useful than usual (1)
- Same as usual (2)
- Less useful than usual (3)
- Much less useful than usual (4)

Q11.4 Have you felt capable of making decisions?

- More than usual (1)
- Same as usual (2)
- Less than usual (3)
- Much less than usual (4)

Q11.5 5. Have you felt constantly overwhelmed and in tension?

- Not at all (1)
- No more than usual (2)
- Somewhat more than usual (3)
- Much more than usual (4)

Q11.6 6. Have you had the feeling that you cannot overcome your difficulties?

- Not at all (1)
- No more than usual (2)
- Somewhat more than usual (3)
- Much more than usual (4)

Q11.7 7. Have you been able to enjoy your normal daily activities?

- More than usual (1)
- Same as usual (2)
- Less than usual (3)
- Much less than usual (4)

Q11.8 8. Have you been able to deal adequately with your problems?

- More than usual (1)
- Same as usual (2)
- Less than usual (3)
- Much less than usual (4)

Q11.9 9. Have you felt unhappy or depressed?

- Not at all (1)
- No more than usual (2)
- Somewhat more than usual (3)
- Much more than usual (4)

Q11.10 10. Have you lost confidence in yourself?

- Not at all (1)
- No more than usual (2)
- Somewhat more than usual (3)
- Much more than usual (4)

Q11.11 11. Have you thought that you are a worthless person?

- Not at all (1)
- No more than usual (2)
- Somewhat more than usual (3)
- Much more than usual (4)

Q11.12 Are you reasonably happy considering all the circumstances?

- More than usual (1)
- Same as usual (2)
- Less than usual (3)
- Much less than usual (4)

Q11.13 Below are a series of questions referring to various aspects of our lives. Please select for each question the number that best expresses your opinion.

1. Do you get the impression that you don't care about the things that are going on around you?

- 1 - Rarely or never (1)
- 2 (2)
- 3 (3)
- 4 (4)
- 5 (5)
- 6 (6)
- 7 - Very often (7)

Q11.14
2. Have you ever been surprised by what people you thought you knew well have done?

- 1 - It has never happened to me (1)
- 2 (2)
- 3 (3)
- 4 (4)
- 5 (5)
- 6 (6)
- 7 - It always happens to me (7)

Q11.15
3. Has it ever happened to you that the people you counted on have let you down?

- 1 - It has never happened to me (1)
- 2 (2)
- 3 (3)
- 4 (4)
- 5 (5)
- 6 (6)
- 7 - It always happens to me (7)

Q11.16
4. So far, your life....

- 1 - Has had no clear objectives or goals at all (1)
- 2 (2)
- 3 (3)
- 4 (4)
- 5 (5)
- 6 (6)
- 7 - Has had very clear objectives and goals (7)

Q11.17
5. Do you feel that you are being treated unfairly?

- 1 - Very often (1)
- 2 (2)
- 3 (3)
- 4 (4)
- 5 (5)
- 6 (6)
- 7 - Rarely or never (7)

Q11.18
6. Do you have the impression of being in a situation that you are not used to and you don't know what to do?

- 1 - Very often (1)
- 2 (2)
- 3 (3)
- 4 (4)
- 5 (5)
- 6 (6)
- 7 - Rarely or never (7)

Q11.19
7. Doing the things you do every day...

- 1 - gives you great joy and satisfaction (1)
- 2 (2)
- 3 (3)
- 4 (4)
- 5 (5)
- 6 (6)
- 7 - causes pain and boredom (7)

Q11.20
8. Do you have very confusing feelings or ideas?

- 1 - Very often (1)
- 2 (2)
- 3 (3)
- 4 (4)
- 5 (5)
- 6 (6)
- 7 - Rarely or never (7)

Q11.21
9. Does it happen to you that you have feelings inside of you that you would rather not have?

- 1 - Very often (1)
- 2 (2)
- 3 (3)
- 4 (4)
- 5 (5)
- 6 (6)
- 7 - Rarely or never (7)

Q11.22
10. Many people, even those with a strong character, sometimes feel like miserable. How many times have you felt this way in your life?

- 1 - Never (1)
- 2 (2)
- 3 (3)
- 4 (4)
- 5 (5)
- 6 (6)
- 7 - Very often (7)

Q11.23
11. When something has happened to you, have you finally seen that...

- 1 - you gave it more or less importance than it really had (1)
- 2 (2)
- 3 (3)
- 4 (4)
- 5 (5)
- 6 (6)
- 7 - you gave things the right importance (7)

Q11.24
12. How many times do you have the impression that the things you do every day mean very little or are of little importance?

- 1 - Very often (1)
- 2 (2)
- 3 (3)
- 4 (4)
- 5 (5)
- 6 (6)
- 7 - Rarely or never (7)

Q11.25
13. How many times do you feel that you are not sure that you can control yourself?

- 1 - Very often (1)
- 2 (2)
- 3 (3)
- 4 (4)
- 5 (5)
- 6 (6)
- 7 - Rarely or never (7)

Group 11: COVID -19 Knowledge

Q12.1 **Finally and to end the survey, answer if the following statements about COVID-19 are true.**

The incubation period for COVID-19 (between infection and the onset of symptoms) is 2 to 14 days.

- Yes (1)
- No (2)
- I don't know (3)

Q12.2
The most common and easily observed symptoms of COVID-19 are fever, dry cough, diarrhea, and shortness of breath.

- Yes (1)
- No (2)
- I don't know (3)

Q12.3 People who have tested positive for COVID-19 must remain in isolation.

- Yes (1)
- No (2)
- I don't know (3)

Q12.4 The main form of transmission is by air (through droplets from people who carry the virus), although it is also transmitted by touching the eyes, nose or mouth after touching contaminated surfaces.

- Yes (1)
- No (2)
- I don't know (3)

Q12.5 COVID-19 begins to spread after symptoms appear.

- Yes (1)
- No (2)
- I don't know (3)

Show this question:

If Are you an active health services professional? = Yes

Q12.6
If the conditions of the centers allow it, it is possible to consider establishing two differentiated circuits: one for patients with respiratory pathology and another for the rest of the users with other reasons for consultation.

- Yes (1)
- No (2)
- I don't know (3)

Show this question:

If Are you an active health services professional? = Yes

Q12.7
If during triage you suspect that you have a case of COVID-19, you must apply the ABCDE as soon as possible.

- Yes (1)
- No (2)
- I don't know (3)

Show this question:

If Are you an active health services professional? = Yes

Q12.8
If the patient presents an acute respiratory infection without criteria for hospital admission and is not part of a vulnerable group, it will be considered a possible case and home isolation will be indicated after performing the diagnostic tests for COVID-19.

- Yes (1)
- No (2)
- I don't know (3)

Show this question:

If Are you an active professional in health services? = yes

Q12.9
Infection should be suspected if 2 or more of the following conditions occur: fever, cough, dyspnea, and staying in a risk area or contact with people diagnosed with COVID-19.

- Yes (1)
- No (2)
- I don't know (3)

Show this question:

If Are you an active health services professional? = Yes

Q12.10
During aerosol-generating procedures, wear tight-fitting full-rimmed eye protection or full face shield, N95 or FFP2 or FFP3 respirator, gloves, and long-sleeved impervious gowns.

- Yes (1)
- No (2)
- I don't know (3)

Show this question:

If Are you an active health services professional? = Yes

Q12.11 Two samples are recommended for the diagnosis of COVID-19: nasopharyngeal and/or oropharyngeal.

- Yes (1)
- No (2)
- I don't know (3)

Show this question:

If Are you an active health services professional? = Yes

Q12.12
In case of transfer of the patient within the out-patient emergency, he must wear a surgical mask.

- Yes (1)
- No (2)
- I don't know (3)

Show this question:

If Are you an active health services professional? = Yes

Q12.13
The radiological picture of those affected by COVID-19 can present from bilateral multifocal opacities that tend to converge, to complete opacification of the lung in the most severe cases.

- Yes (1)
- No (2)
- I don't know (3)

Show this question:

If Are you an active professional in health services? = yes

Q12.14 In the case of pregnant women over 24 weeks, fetal well-being will be checked and the patient will remain under observation until the result of the diagnostic tests. Fetal well-being will be monitored by ultrasound and/or cardiotocographic recording (RCTG) depending on the weeks of pregnancy.

- Yes (1)
- No (2)
- I don't know (3)

Show this question:

If Are you an active professional in health services? = yes

Q12.15
COVID-19 cases usually present with very serious symptoms.

- Yes (1)
- No (2)
- I don't know (3)

Q13.1 SUGGESTIONS

If you have any suggestion, recommendation and/or query, or wish to reflect on any issue that you have not been able to indicate in the development of the questionnaire, please, do so below:

________________________________________________________________

________________________________________________________________

________________________________________________________________

________________________________________________________________

________________________________________________________________

Q13.2 Finally, if you are interested in participating in future related studies, please, state your email and/or contact telephone number:

Email address (4) __________________________________________________

Telephone (5) __________________________________________________
